# Cardiac PET/CT with Rb-82: optimization of image acquisition and reconstruction parameters

**DOI:** 10.1186/s40658-017-0178-3

**Published:** 2017-02-15

**Authors:** P. Chilra, S. Gnesin, G. Allenbach, M. Monteiro, J. O. Prior, L. Vieira, J. A. Pires Jorge

**Affiliations:** 1 Haute École de Santé Vaud - Filière TRM, University of Applied Sciences and Arts Western Switzerland, Lausanne, Switzerland; 2 Institute of Radiation Physics, Lausanne University Hospital, Lausanne, Switzerland; 3 Nuclear Medicine and Molecular Imaging Department, Lausanne University Hospital, Lausanne, Switzerland; 4 Área Científica de Medicina Nuclear, Escola Superior de Tecnologia da Saúde de Lisboa, Instituto Politécnico de Lisboa, Lisbon, Portugal; 5 Instituto de Biofísica e Engenharia Biomédica, Faculdade de Ciências da Universidade de Lisboa, Lisbon, Portugal

**Keywords:** Rb-82, Myocardial blood flow, Myocardial flow reserve, Cardiac PET/CT reconstruction

## Abstract

**Background:**

Our aim was to characterize the influence of time-of-flight (TOF) and point spread function (PSF) recovery corrections, as well as ordered subset expectation maximization (OSEM) reconstruction parameters, in ^82^Rb PET/CT quantification of myocardial blood flow (MBF) and myocardial flow reserve (MFR).

Rest and stress list-mode dynamic ^82^Rb PET acquisition data from 10 patients without myocardial flow defects and 10 patients with myocardial blood flow defects were reconstructed retrospectively. OSEM reconstructions were performed with Gaussian filters of 4, 6, and 8 mm, different iterations, and subset numbers (2 × 24; 2 × 16; 3 × 16; 4 × 16). Rest and stress global, regional, and segmental MBF and MFR were computed from time activity curves with FlowQuant^©^ software. Left ventricular segmentation using the 17-segment American Heart Association model was obtained.

**Results:**

Whole left ventricle (LV) MBF at rest and stress were 0.97 ± 0.30 and 2.30 ± 1.00 mL/min/g, respectively, and MFR was 2.40 ± 1.13. Concordance was excellent and all reconstruction parameters had no significant impact on MBF, except for the exclusion of TOF which led to significantly decreased concordance in rest and stress MBF in patients with or without perfusion defects on a coronary artery basis and in MFR in patients with perfusion defects.

**Conclusions:**

Changes in reconstruction parameters in perfusion ^82^Rb PET/CT studies influence quantitative MBF analysis. The inclusion of TOF information in the tomographic reconstructions had significant impact in MBF quantification.

## Background

Hybrid cardiac positron emission tomography/computed tomography (PET/CT) with ^82^rubidium (^82^Rb) is a useful clinical tool for the diagnosis of unknown or suspected coronary arterial disease (CAD) and microvascular disease, improving the understanding of the pathophysiology of disease processes thus directly impacting the therapeutic approach [1–3]. This short-lived radioisotope (half-life 76.4 s) is a monovalent cationic analog of potassium produced by a ^82^strontium/^82^rubidium (^82^Sr/^82^Rb) generator, allowing clinical imaging with short protocols (20–30 min in total), providing better results than conventional SPECT techniques with improved values of sensitivity and specificity in the order of 90% [4–6]. The main reasons for improved diagnostic performance are the radionuclide (^82^Rb) kinetic properties, better resolution and sensitivity PET scanners, and the accurate attenuation correction (AC) achievable in modern PET/CT imaging [6–8]. Furthermore, the use of cardiac ^82^Rb PET/CT has been promoted by its ability to quantify myocardial blood flow (MBF) and myocardial flow reserve (MFR) noninvasively and routinely [9, 10]. Accuracy and precision in quantitative MBF ^82^Rb dynamic PET imaging have been recently reviewed [11] while reproducible results can be achieved with the available software [12].Improvement of PET quantitative cardiac imaging can be obtained by optimization of acquisition parameters [13–15]. Present generation PET/CT includes time-of-flight (TOF) information to better recover the localization of positron annihilations sites [13], with consequent statistical noise reduction and increase in lesion contrast [16–18] and benefits in ^82^Rb PET perfusion studies of obese patients [19]. Furthermore, this method seems less sensitive to mismatched attenuation correction, erroneous normalization, and poorly estimated scatter correction, but such robustness also depends on the time resolution of the TOF-PET scanner [16–18, 20, 21]. Recently, tomographic reconstruction algorithms including point spread function (PSF) recovery became available. PSF inclusion in the iterative tomographic reconstruction process leads to valuable improvements of spatial resolution with consequent reduction of partial volume effects (PVE) translating in increased activity recovery and lesion contrast [22]. Because spurious events may also appear erroneously in regions of the image where there is no true activity, the implementation of robust scatter and attenuation corrections appears essential [17, 23–25]. On the other hand, the quantification of MBF and MFR in ^82^Rb PET images might also be affected by reconstruction type (iterative or analytical). Cardiac PET image reconstruction can be achieved by both filtered back projection (FBP) and iterative reconstruction algorithms [26]. The filtered back projection (FBP) method is fast, linear, and robust and often still is the preferred reconstruction method for dynamic PET studies consisting of many frames with short scan durations and thus poor statistics [27–29]. However, reconstructed images by FBP may contain severe streak artifacts, increasing image noise and reducing contrast, which may mask lesions [27–29]. Thus, FBP has been replaced by iterative algorithms in oncological PET, as their reconstruction times became clinically acceptable. Consequently, the principal method used is the iterative ordered subset expectation maximization (OSEM), which processes the projection data subset by subset instead of all the data at once [27]. The aim of this study was to investigate the quantitative influence of TOF and PSF recovery corrections, as well as reconstruction parameters (subsets × iteration product; filters) used in OSEM algorithms for cardiac ^82^Rb PET/CT, as a first step towards optimization or recommendations.

## Methods

### Study population

Twenty adult patients were referred for cardiac ^82^Rb PET/CT and underwent rest and adenosine stress studies at the Lausanne University Hospital. The data included in this study were retrospectively collected between September and December 2013. Patients were divided into two groups: G_1_ (*n* = 10 patients without myocardial flow defects) and G_2_ (*n* = 10 patients with myocardial flow abnormalities, such as sequels of myocardial infarction (*n* = 2; 20%), stress ischemia (*n* = 3; 30%, SDS ≥3), or globally diminished myocardial flow reserve (*n* = 5; 50%)). Patient characteristics are described in Table 1.

**Table 1:** Patient characteristics according to the group without and with myocardial flow defects (mean ± SD or *n* (%)) according to standard reconstruction protocol (protocol A in Table 2)

	Patients without myocardial flow defects (*n* = 10)	Patients with myocardial flow defects (*n* = 10)
Age (years)	62 ± 9	68 ± 9
Sex (female/male)	3/7 (30%/70%)	2/8 (20%/80%)
BMI (kg/m^2^)	27 ± 6	29 ± 7
Hypertension	5 (50%)	5 (50%)
Diabetes mellitus	2 (20%)	3 (30%)
Dyslipidemia	3 (30%)	3 (30%)
Current smokers	2 (20%)	0 (0%)
Prior myocardial infarction	0 (0%)	2 (20%)
Ischemia	0 (0%)	3 (30%)
Decreased MFR	0 (0%)	5 (50%)
LV rest MBF (mL/min/g)	1.10 ± 0.31	0.84 ± 0.24
LV stress MBF (mL/min/g)	3.08 ± 0.58	1.50 ± 0.63
LV MFR [1]	3.03 ± 1.22	1.76 ± 0.51

*BMI* body mass index, *LV* left ventricle, *MBF* myocardial blood flow, *MFR* myocardial flow reserve

### Patient preparation

Written informed consent was obtained for all patients before inclusion, and history taking covering symptoms, risk factors, diseases, medication, and prior diagnostic or therapeutic procedures was performed. Patients were refrained from taking caffeine-containing substances for at least 24 h and were asked to fast at least 6 h prior to the PET studies; medications that may interfere with the cardiac pharmacological stress agent (e.g., nitrates or beta-blockers) were suspended for ≥24 h.

### Protocol and image acquisition

All dynamic studies were performed using a 3D PET/CT scanner (Discovery 690; GE Healthcare, Waukesha, WI) equipped with cerium-doped lutetium yttrium orthosilicate (LYSO) scintillation crystals (13824 LYSO crystals of size: 4.2 × 6.3 × 25 mm2); axial field of view (FOV) is 157 mm while transaxial FOV is 70 cm. Annihilation photons were acquired in a 425–650 keV energy window. TOF coincidence-timing resolution is 550 ps. Emission images at rest were obtained after ^82^RbCl administration of 10 MBq/kg (max 1300 MBq) by an automated system (^82^Sr/^82^Rb generator). Pharmacological stress was induced with adenosine (140 μg/kg/min) to obtain stress perfusion results. A second injection of radiopharmaceutical (10 MBq/kg) was injected ≥10.1 min after the first injection, at the end of the second minute of adenosine intravenous infusion. A list-mode acquisition was started simultaneously with radiopharmaceutical injection for both studies (rest, stress), and dynamic images were reconstructed (21 frames, 35 slices each; 12 × 8, 5 × 12, 1 × 30, 1 × 60, 1 × 120, and 1 × 240 s) for deriving absolute flow measurement. The attenuation maps for rest and stress studies were obtained from the imbedded 64-slice CT scanner adopting a low-dose CT setup (120 kV, 10 mA, 0.8 s per rotation, pitch 1.375:1). The first CT was acquired after the scout scan for accurate definition of the axial examination range and just before the rest study; the second CT scan was repeated immediately after the stress acquisition. All stress procedures were supervised by a qualified physician with knowledge of pharmacological stress agents and expertise in advanced life support techniques.

### OSEM reconstruction protocols

Reconstruction protocols (RP) with Gaussian filters of 4, 6, and 8 mm and variable iterations and subsets (iterations × subsets) of 2 × 24, 2 × 16, 3 × 16, and 4 × 16 were created, as described in the Table 2. A total of 320 reconstructions were performed, 20 for each patient (including rest and stress dynamic studies). The standard reference RP used in the nuclear medicine department for dynamic studies with ^82^Rb was OSEM + TOF with OSEM reconstruction 2 iterations × 24 subsets and a full-width half maximum (FWHM) filter of 6 mm. In the absence of a quantitative “gold standard,” to investigate the influence of reconstruction parameters (TOF, PSF, iterations ×subsets, and filters) in the quantification of MBF and MFR, we compared results obtained from the tested RPs with our standard reference protocol.

**Table 2:** Reconstruction protocols (changes to standard protocol are marked in italics)

Reconstruction protocols	Iterations × subsets	Filter FWHM (mm)	Difference vs. standard protocol (A)
(A) OSEM + TOF	2 × 24 = 48	6	–
(B) OSEM + TOF	4 × 16 = *64*	6	Higher iterations × subsets product
(C) OSEM + TOF	3 × 16 = 48	*4*	Lower smoothing
(D) OSEM + TOF	2 × 16 = *32*	6	Lower iterations × subsets product
(E) OSEM + TOF + *PSF*	3 × 16 = 48	6	With PSF recovery
(F) OSEM + TOF	3 × 16 = 48	*8*	Higher smoothing
(G) OSEM + TOF	*3* × *16* = 48	6	Same product of iterations × subsets, but different iteration and subset
(H) OSEM + no TOF	3 × 16 = 48	6	No TOF correction

*OSEM* ordered subsets expectation maximization, *TOF* time of flight, *PSF* point spread function

### Image reconstruction and processing

About 14% of ^82^Rb β + decays also produce a 776.5 keV γ-ray with no angular correlation. This results in a significant pollution of the true coincidence emission data, distinct in shape from the scatter component, and must be corrected in view of quantitative cardiac perfusion imaging [27, 30, 31]. Quantitative PET reconstructions were corrected for prompt gamma pollution using a specific correction method provided by the vendor (not yet reported in the literature). Image quality control (QC) was used to obtain a precise alignment of the heart in PET and CT images in the short axis, vertical, and horizontal long-axis views. Rest and stress dynamic studies were reconstructed from list mode.

### Quantitative analysis of MBF and MFR

^82^Rb time activity curves (TACs) were used to generate automatically segmental, regional, and global MBF, MFR (stress/rest), and myocardial flow difference (stress-rest) polar maps using FlowQuant© software (Ottawa Heart Institute, Ottawa, CA) [32, 33]. According to the American Heart Association (AHA) recommendations for the left ventricle (LV) segmentation [34], each polar map (rest, stress) was divided into 17 different segments corresponding to the LV myocardium, which can be assigned to one of the three coronary artery territories. A 1-tissue compartment modeling procedure was used to calculate MBF and MFR in absolute terms (mL/min/g) [31–33]. This model included an input function derived from arterial blood in the base of the left ventricle, regional uptake and clearance parameters (*K*_1_, mL/min/g and *k*_2_, min^−1^), blood to myocardium spillover fraction *f*_*b*_, and myocardial partial volume correction (1−*f*_*b*_). The ^82^Rb extraction fraction was used to estimate MBF and consequently MFR (stress/rest) from *K*_1_. The activity concentration in the myocardium *C*_m_ (*t*) was calculated by:

$$ {C}_m(t)={K}_1\cdot {e}^{- k2\cdot 1}\otimes {C}_{\mathrm{LV}}(t), $$

where *K*_1_ and *k*_2_ represent the uptake and washout constants and *C*_LV_(*t*) the activity concentration of the blood in the LV, and ⊗ represents the convolution operator.

^82^RbCl does not accumulate in/or clear from myocardium proportionally linear to the perfusion caused by an incomplete and curvilinear myocardial extraction fraction from arterial blood with increasing flow rates [8]. Parametric images representing a graphical illustration of quantitative MBF were then generated to display perfusion at the voxel level, based on the evaluation of the tracer kinetic model for each voxel [33]. Voxels were also grouped to give MBF according to each of the 17 AHA segment and then according to coronary artery territories and finally the whole LV [34].

### Statistical methods

The differences to the standard clinical reconstruction protocol were analyzed using Lin’s concordance correlation coefficient and the Bland-Altman (BA) plots [35, 36]. In Lin’s concordance coefficient *ρ*_c_ is considered as a measure of precision and accuracy to assess the degree of agreement between two measures of the same variables performed with different reconstruction parameters [35]. In all cases, a perfect concordance gives *ρ*_c_ = 1 and the absence of concordance *ρ*_c_ = 0. This approach offers several advantages to compare measurements of the same value and has been used to compare MBF measurements obtained from several software packages [32]. Box plots with median and quartile (upper and lower) values were created for rest, stress, and reserve results of all RP. For BA analysis, we calculated the mean (*x*-axis) and the differences (*y*-axis) between each RP comparison to obtain a graph to evaluate the discrepancy of values in the rest and stress dynamic studies.

The 95% confidence interval limits of concordance were included in all graphs and tables. Using these limits, it is easily seen if two concordance coefficients are alike or different at the *p* < 0.05 level just by checking for the presence of overlapping 95% CI limits (no difference between methods as concordance coefficients or *p* > 0.05) or not (significant difference between methods with *p* ≤ 0.05).

All statistical analyses were performed with Stata 13.0 software (Stata Corporation, College Station, TX) using a *p* value <0.05 as the significance level.

## Results

**Fig. 1:**
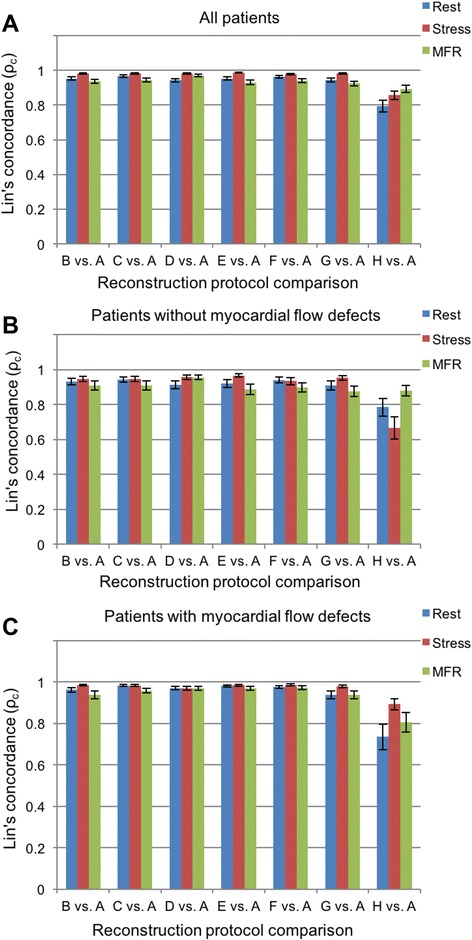
Concordance coefficients *ρ*_c_ for rest, stress MBF, and myocardial flow reserve (MFR) for: **a** all patients, **b** patients without myocardial flow defects, and **c** patients with myocardial flow defects in segmental analysis

Lin’s concordance coefficients for rest, stress MBF, and MFR in the three coronary artery territories and 17 segments, as shown in Tables 3 and 4, respectively, as well as in Tables 5 and 6, according to the presence or absence of perfusion abnormalities (Figure 1). The difference in values between each RP and standard setting was used to identify which changes to the reconstruction parameters affected the MBF or MFR the most, based on the overlapping or not of the 95% CI given in the tables, defining whether any comparison was significant at the *p* < 0.05 value level.

**Table 3:** Lin’s concordance coefficient *ρ*_c_ obtained for MBF and MFR comparisons in the three coronary territories (LAD, LCX, RCA)

RP comparisons	All patients (*n* = 60 coronary territories)		
	*ρ*_c (Rest)_ [95% CI]	*ρ*_c (Stress)_ [95% CI]	*ρ*_c (MFR)_ [95% CI]
B vs. A	*0.955* [0.933;0.977]	*0.983* [0.975;0.992]	0.939 [0.910;0.967]
C vs. A	*0.966* [0.949;0.982]	*0.984* [0.976;0.992]	0.909 [0.847;0.972]
D vs. A	*0.939* [0.836;0.959]	*0.985* [0.977;0.992]	0.957 [0.928;0.987]
E vs. A	*0.951* [0.927;0.976]	*0.990* [0.985;0.995]	0.883 [0.808;0.958]
F vs. A	*0.968* [0.954;0.982]	*0.984* [0.976;0.992]	0.898 [0.836;0.960]
G vs. A	*0.938* [0.909;0.967]	*0.986* [0.980;0.992]	0.876 [0.807;0.845]
H vs. A	0.769* [0.678;0.861]	0.789* [0.789;0.912]	0.880 [0.805;0.955]

*p < 0.05 vs. other comparisons (in italics in same column)

**Table 4:** Lin’s concordance coefficient *ρ*_c_ obtained for MBF and MFR comparisons in the 17-segment model

RP comparisons	All patients (*n* = 340 segments)		
	*ρ*_c (Rest)_ [95% CI]	*ρ*_c (Stress)_ [95% CI]	*ρ*_c (MFR)_ [95% CI]
B vs. A	*0.954* [0.945;0.964]	*0.982* [0.978;0.986]	*0.938* [0.926;0.950]
C vs. A	*0.966* [0.959;0.973]	*0.982* [0.978;0.986]	*0.945* [0.933;0.956]
D vs. A	*0.943* [0.933;0.954]	*0.982* [0.978;0.986]	*0.971* [0.965;0.977]
E vs. A	*0.954* [0.944;0.964]	*0.988* [0.985;0.990]	*0.931* [0.918;0.944]
F vs. A	*0.962* [0.955;0.970]	*0.980* [0.976;0.984]	*0.941* [0.930;0.952]
G vs. A	*0.944* [0.933;0.955]	*0.983* [0.980;0.987]	0.926 [0.912;0.939]
H vs. A	0.794* [0.759;0.829]	0.857* [0.833;0.882]	0.893* [0.872;0.914]

*p < 0.05 vs. other comparisons (in italics in same column)

**Table 5:** Lin’s concordance coefficient *ρ*_c_ obtained for MBF and MFR comparisons in the three coronary territories (LAD, LCX, RCA)

RP comparisons	Patients without myocardial flow defects (*n* = 30 territories)			Patients with myocardial flow defects (*n* = 30 territories)		
	*ρ*_c (Rest)_ [95% CI]	*ρ*_c (Stress)_ [95% CI]	*ρ*_c (MFR)_ [95% CI]	*ρ*_c (Rest)_ [95% CI]	*ρ*_c (Stress)_ [95% CI]	*ρ*_c (MFR)_ [95% CI]
B vs. A	0.922 [0.873;0.971]	*0.931* [0.888;0.974]	0.904 [0.838;0.969]	*0.971* [0.951;0.991]	*0.986* [0.976;0.996]	*0.951* [0.917;0.985]
C vs. A	*0.935* [0.893;0.977]	*0.937* [0.897;0.977]	0.909 [0.847;0.972]	*0.989* [0.982;0.996]	*0.984* [0.974;0.995]	*0.962* [0.938;0.986]
D vs. A	*0.897* [0.836;0.959]	*0.949* [0.914;0.984]	0.957 [0.928;0.987]	*0.973* [0.954;0.991]	0.968 [0.947;0.989]	*0.975* [0.956;0.993]
E vs. A	0.907 [0.843;0.970]	*0.963* [0.939;0.987]	0.883 [0.808;0.958]	*0.985* [0.975;0.995]	*0.985* [0.974;0.996]	*0.972* [0.952;0.992]
F vs. A	*0.948* [0.919;0.977]	*0.925* [0.873;0.978]	0.898 [0.836;0.960]	*0.978* [0.964;0.993]	*0.987* [0.979;0.995]	*0.976* [0.959;0.993]
G vs. A	0.889 [0.817;0.961]	*0.947* [0.910;0.985]	0.876 [0.807;0.845]	*0.977* [0.961;0.993]	*0.978* [0.964;0.993]	*0.944* [0.905;0.983]
H vs. A	0.752* [0.618;0.887]	0.606* [0.446;0.766]	0.880 [0.805;0.955]	0.689* [0.522;0.856]	0.884* [0.811;0.957]	0.766* [0.627;0.904]

*p < 0.05 vs. other comparisons (in italics in same column)

**Table 6:** Lin’s concordance coefficient *ρ*_c_ obtained for MBF and MFR comparisons in the 17-segment model

RP comparisons	Patients without myocardial flow defects (*n* = 170 segments)			Patients with myocardial flow defects (*n* = 170 segments)		
	*ρ*_c (Rest)_ [95% CI]	*ρ*_c (Stress)_ [95% CI]	*ρ*_c (MFR)_ [95% CI]	*ρ*_c (Rest)_ [95% CI]	*ρ*_c (Stress)_ [95% CI]	*ρ*_c (MFR)_ [95% CI]
B vs. A	*0.932* [0.913;0.951]	*0.945* [0.930;0.959]	0.908 [0.882;0.934]	*0.962* [0.951;0.973]	*0.985* [0.980;0.989]	*0.936* [0.918;0.955]
C vs. A	*0.943* [0.927;0.958]	*0.945* [0.930;0.960]	0.909 [0.884;0.935]	*0.983* [0.978;0.988]	*0.983* [0.978;0.988]	*0.957* [0.946;0.969]
D vs. A	*0.911* [0.889;0.934]	*0.956* [0.943;0.968]	0.954 [0.941;0.967]	*0.970* [0.962;0.978]	*0.969* [0.960;0.977]	*0.968* [0.959;0.977]
E vs. A	*0.920* [0.898;0.943]	*0.966* [0.957;0.976]	0.886 [0.856;0.917]	*0.980* [0.974;0.986]	*0.984* [0.979;0.989]	*0.968* [0.959;0.978]
F vs. A	*0.940* [0.924;0.956]	*0.933* [0.913;0.952]	0.897 [0.871;0.924]	*0.976* [0.970;0.983]	*0.986* [0.982;0.990]	*0.973* [0.964;0.981]
G vs. A	*0.907* [0.882;0.933]	*0.952* [0.938;0.966]	0.874 [0.845;0.904]	*0.938* [0.920;0.956]	*0.978* [0.971;0.984]	*0.938* [0.920;0.956]
H vs. A	0.784* [0.734;0.834]	0.667* [0.604;0.730]	0.879 [0.848;0.909]	0.735* [0.673;0.796]	0.892* [0.864;0.920]	0.805* [0.757;0.853]

*p < 0.05 vs. other comparisons (in italics in same column)

**Fig. 2:**
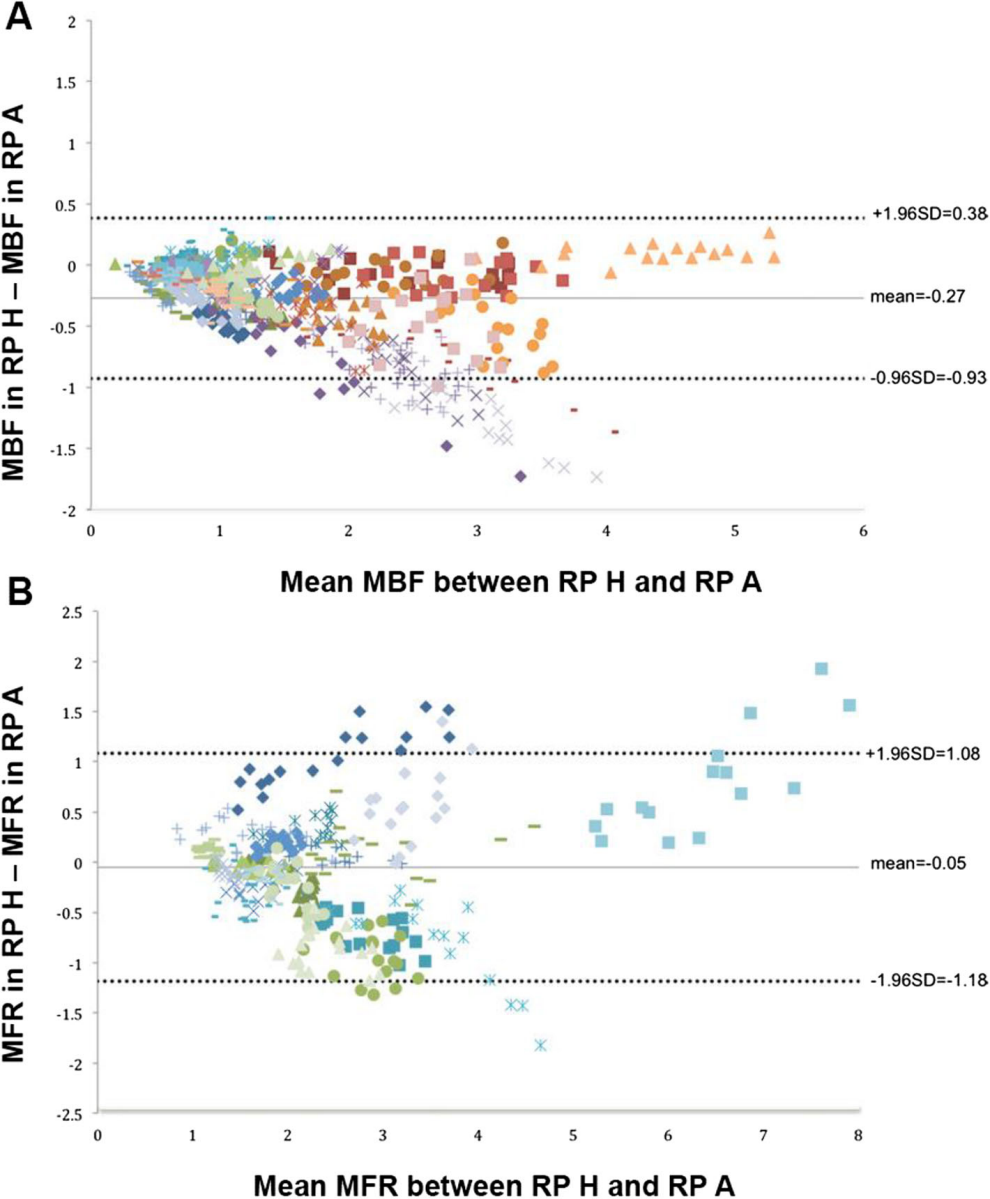
BA plot for rest and stress (**a**) MBF and **b** MFR differences between RP H and RP A (standard). Simplified legend: Each individual shape represents a separate patient; *warm tones* correspond to stress MBF and *cold tones* to rest MBF or MFR

**Fig. 3:**
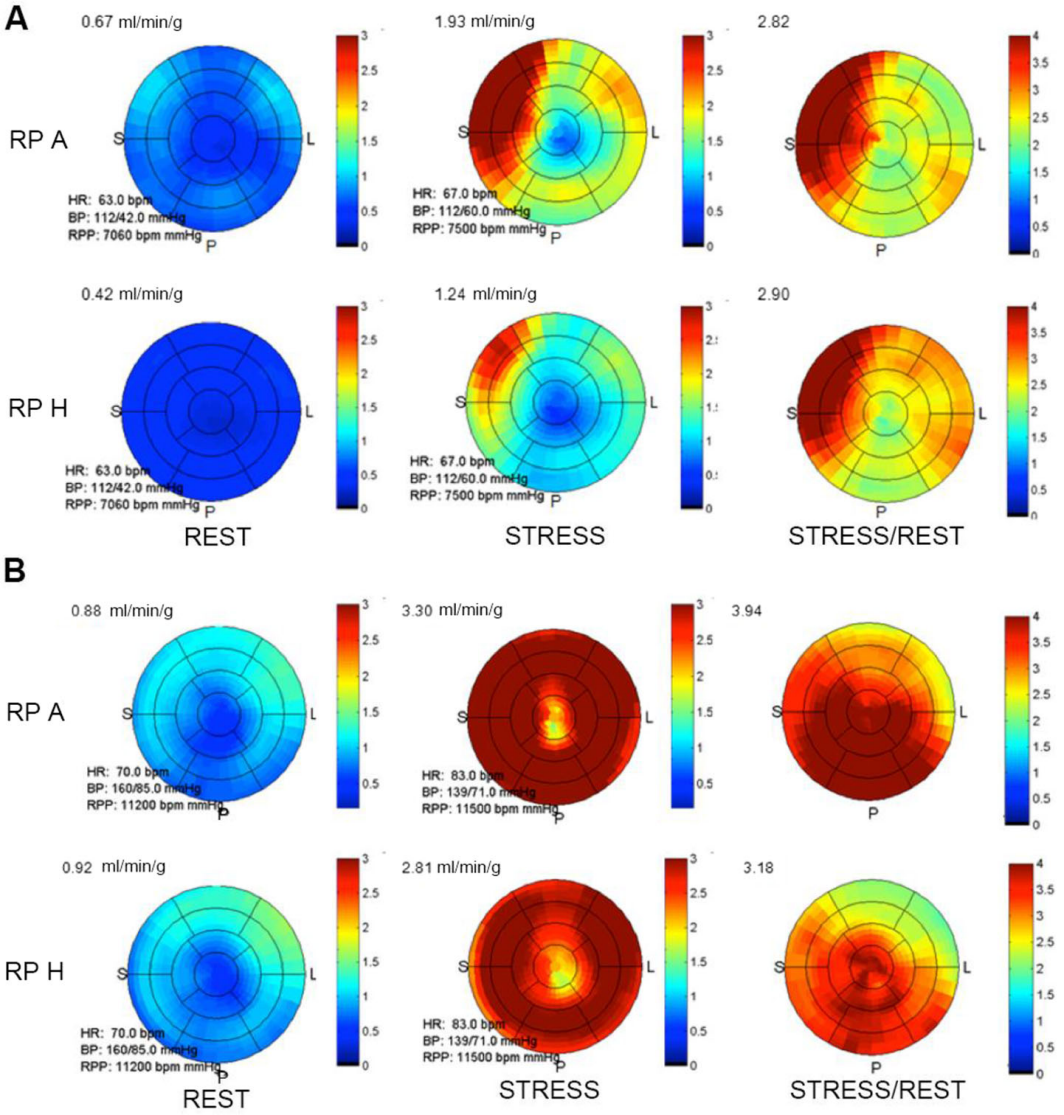
Polar maps for MBF (rest and stress) and MFR. **a** Patient with myocardial flow defects (stress ischemia). **b** Patient without myocardial flow defects

**Fig. 4:**
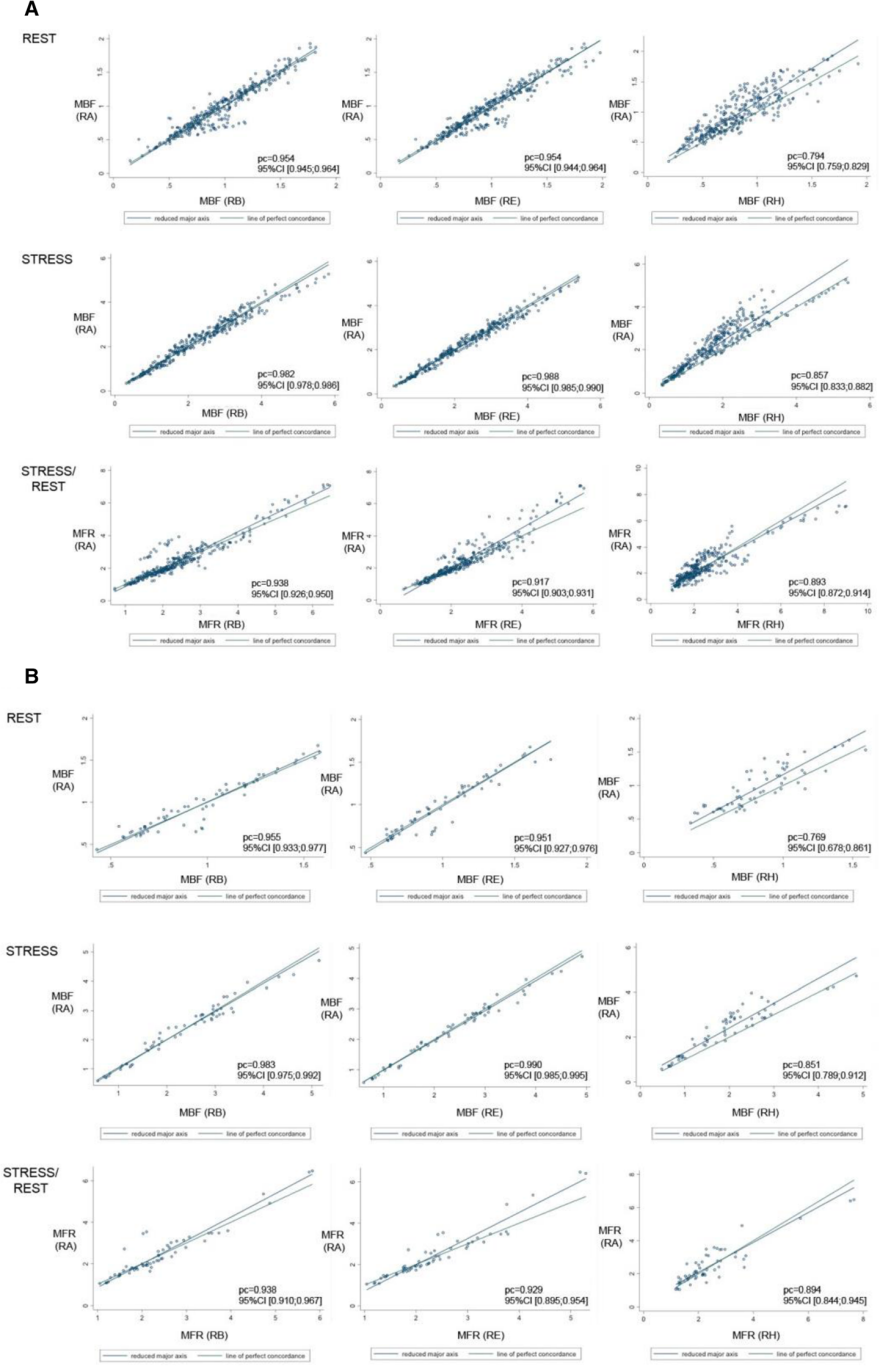
Lin’s concordance coefficient graphs for (**a**) segmental and **b** regional analyses between RP B, E, H, and RP A (standard) in all patients. Rest and stress MBF and MFR (stress MBF/rest MBF) are represented

The results of comparisons between the different combination of RP and the standard protocol used (RP A: OSEM + TOF; 2 iterations × 24 subsets, FWHM = 6 mm) showed a good correlation in the majority of cases, with concordance values close to *ρ*_c_ = 1.0. However, the concordance was significantly lower for the RP H without TOF in the rest and stress MBF for patient groups G_1_ and G_2_ in the coronary artery territories (*p* < 0.05, Table 5) and in the 17-segment evaluation (*p* < 0.05, Table 6). The statistical analyses applied to MFR results for G_2_ also reveal significant differences for the same RP without TOF (protocol H). On the other hand, MFR values for G_1_ exhibited no significant difference compared to the reference standard RP on a coronary territory level (Table 5), while it was significant on the 17-segment level (Table 6). BA plots (Figure 2) depict the differences in rest and stress data reconstructed by protocols A and H. The largest changes could be observed once more in reconstruction without TOF (Figure 3). Similar differences existed when comparing protocol G vs. protocol H, which only differed by the removal of TOF (data not shown). In contrast, both rest and stress studies reconstructed with OSEM + TOF + PSF showed less dispersion when compared to the standard protocol (Figure 4). Changes in iteration and subset numbers showed to have minimal influence on MBF in contrary to removal of TOF. In individual segments, these changes could reach up to 1.7 mL/min/g.

## Discussion

Our investigations were performed to evaluate the effect of reconstruction parameters and different algorithms in quantitative cardiac ^82^Rb PET/CT imaging. Over the years, changes in reconstruction parameters have been made mainly for ^18^F-FDG PET/CT and can be considered crucial for improving image quality. However, few studies have been specifically performed in cardiac PET imaging to study the influence of reconstruction parameters on image reconstruction quality in clinically relevant condition with ^82^Rb.

The benefit arising from the combined use of TOF and PSF corrections in quantitative cardiac PET has been investigated by Presotto et al. [37]; this study was limited to only a phantom setup filled with ^18^F-FDG. Because of the short half-life of ^82^Rb and the relative high activity to handle, realistic phantom studies in clinical relevant condition are not easy to perform. Nevertheless, recently, Renaud at al. [38] tested the MBF quantitative accuracy in ^82^Rb cardiac PET in different PET scanners. In particular, the specific activity administered to patients in our study (10 MBq/kg) is very close to the optimal value (11.4 MBq/kg) found by Renaud et al. for the employed PET scanner (GE Discovery 690). Thus, we do not expect quantitative bias due to the total activity of radiotracer administered to patients, especially at the fist pass bolus transit.

In our study, different RPs were created and compared with a reference standard protocol used routinely in our nuclear medicine department, to understand the impact of TOF and PSF corrections included in OSEM reconstruction algorithms. The influence of the number of iterations, subsets, and Gaussian filtering on MBF and MFR quantification was also assessed. The number of subsets used in RP was 16 or 24 to avoid image degradation (i.e., noise, artifacts), as PET image quality with OSEM declined as the number of subsets increased, even when the TOF algorithm was used [39].

Rest and stress dynamic studies reconstructed without TOF showed a significant difference in global and regional MBF values, comparatively to the standard protocol, in G_1_ and G_2_. This can be explained because TOF algorithm is crucial to improve image quality and lesion contrast potentially providing more reliable results. The largest differences in MBF values, in relation to the reference standard protocol, appeared in the RP without TOF correction. Maebatake et al. used phantom studies based on the relationship between the noise equivalent count (NEC), as an index of PET image quality and TOF-PET image quality for ^18^F-FDG PET/CT showing similar results regarding the impact of PSF and TOF [40]. Significant improvements on image contrast were observed in the OSEM reconstructions combined with the PSF algorithm. The study also highlighted that similar coefficients of variance are obtained for nonTOF and TOF reconstructions; however, a longer acquisition time is required for those without TOF to obtain identical image contrast and reduce levels of noise [40]. For the TOF + PSF RPs, no major differences were revealed from the standard, presenting both high *ρ*_c_ values, which means a good correlation between two study variables. According to the results of Akamatsu et al. [41], the combination TOF + PSF in ^18^F-FDG PET/CT clearly improves image quality, showing a more uniform background. In our study, we did not observe a significant difference by adding PSF recovery; we can hypothesize that this might not have a big effect as the heart is moving and thus blurring the image anyway. A few patients had small hearts, which can increase the spillover effect (due to the proximity of blood pool volume of interest and myocardium) and underestimate MBF values obtained for both groups of patients in OSEM reconstruction.

The present study had some limitations. First, the number of patients included in this study could have been bigger in order to improve the statistical pertinence of the presented results. Second, a phantom study providing gold standard values for MBF and MFR (normal database) would have been beneficial but very difficult to perform with such a so short-lived radiotracer. Third, there might have been the presence of patient with high stress flows and reserve, who might have biased our results, but as this was real patient data, we decided not to exclude them as they are observed in clinical practice. Fourth, it is also difficult to evaluate whether the observed differences have a true clinical significance or not. As pinpointed by Moody et al., statistical uncertainty in the Renkin-Crone relation account for 50–70% of the coefficient of variation at rest and 40–50% at stress [11], so that we can only evaluate if the different parameters produce similar or discrepant results. Fifth, the comparison of concordance coefficient between G_1_ and G_2_ might have been influenced by the dynamic range of MBF and MFR.

Furthermore, a RP with OSEM + PSF without TOF would be interesting to study for understanding the role of PSF correction alone in the quantification of MBF and MFR.

Finally, additional prospective investigations with FBP algorithm are warranted to explore the main differences between analytical and iterative reconstructions in ^82^Rb dynamic PET/CT measurements.

## Conclusions

Changes in reconstruction parameters applied to rest and stress ^82^Rb perfusion studies influenced MBF and MFR quantitation. Concordance among the different reconstruction protocols was excellent and most variations had little effect, except for the exclusion of TOF, which significantly reduced concordance of rest and stress MBF, as well as MFR, both at the coronary territory and segmental level.

## Abbreviations

^82^Rb: Rubidium-82
^82^Sr/^82^Rb: Strontium-82/Rubidium-82
AC: Attenuation correction
BA: Bland-Altman
CAD: Coronary arterial disease
FBP: Filtered back projection
FOV: Field of view
FWHM: Full-width half maximum
LV: Left ventricle
LYSO: Lutetium yttrium orthosilicate
MBF: Myocardial blood flow
MFR: Myocardial blood reserve
NEC: Noise-equivalent count
OSEM: Ordered subset expectation maximization
PET/CT: Positron emission tomography/computed tomography
Prompt: Gamma
PSF: Point spread function
PVE: Partial volume effects
QC: Quality control
RP: Reconstruction protocol
SPECT: Single photon emission computerized
TACs: Time activity curves
TOF: Time of flight
